# Rapid improvement in vitamin D status with dietary 25-hydroxycholecalciferol in vitamin D insufficient dogs

**DOI:** 10.1017/jns.2021.4

**Published:** 2021-02-22

**Authors:** Rachel A. Kurzbard, Robert C. Backus, Shiguang Yu

**Affiliations:** 1 Department of Veterinary Medicine and Surgery, College of Veterinary Medicine, University of Missouri, Columbia, MO 65211, USA; 2 DSM Nutritional Products, LLC, Parsippany, NJ 07054, USA

**Keywords:** Canine, Calcifediol, Cholecalciferol, 25-hydroxycholecalciferol, HyD^®^

## Abstract

Vitamin D insufficiency is associated with various disease processes. We determined whether consumption of a diet supplemented with HyD^®^, a 25-hydroxycholecalciferol (25(OH)D_3_) source, would safely increase plasma 25(OH)D_3_ concentrations in Golden Retrievers with low vitamin D status. We hypothesised that dietary supplementation with HyD^®^ would rapidly increase and sustain plasma 25(OH)D_3_ levels in healthy Golden Retrievers with low vitamin D status compared with supplementation with vitamin D_3_. Of fifty-seven privately owned dogs recruited with written owner consent, eighteen dogs with low vitamin D status were identified and sorted between two groups to have similar initial plasma 25(OH)D_3_ concentrations, sex distributions, ages and body weights. Dogs of each group were fed a dry dog food supplemented with either 16 μg/kg of 25(OH)D_3_ as HyD^®^ (*n* 10) or 81 μg/kg of cholecalciferol (D_3_) (*n* 8) for 4 months. Plasma 25(OH)D_3_ concentrations were determined monthly. A significant time effect (*P* < 0⋅001) and time by group interaction (*P* = 0⋅0045) were found for monthly determined plasma 25(OH)D_3_ concentrations. Dogs fed the HyD^®^-supplemented diet experienced a 40⋅5 % rise in plasma 25(OH)D_3_ values after 1 month (*P* < 0⋅001) and no change thereafter. Plasma 25(OH)D_3_ values of dogs supplemented with vitamin D_3_ did not increase (*P* > 0⋅05) and were less than values of dogs supplemented with HyD^®^ (*P* = 0⋅044). With few exceptions, average haematologic, biochemical and urinalyses results remained within the reference range for both groups. Dietary supplementation with HyD^®^ is sufficient to safely increase and sustain plasma 25(OH)D_3_ levels in healthy dogs.

## Introduction

Vitamin D is essential for numerous metabolic functions in dogs and other species. Unlike man, dogs are unable to use ultraviolet rays to synthesise sufficient vitamin D in the skin to meet their requirement^(1)^. Dogs rely on their diet to fulfil the vast majority of their vitamin D requirement. Vitamin D is most commonly known for its role in maintaining calcium and phosphorus homeostasis. Vitamin D also plays numerous key roles via receptors on target cells to modulate immune and cardiovascular function^(2–4)^.

The best indicator of vitamin D status is plasma 25-hydroxyvitamin D (25(OH)D), which is the most abundant circulating metabolite of vitamin D^(4)^. Adequate intake levels of vitamin D in dogs were recommended by the National Research Council (NRC), based on the amount required to prevent bone pathologies in growing puppies^(5)^. These values have been extended to adult dogs. However, the vitamin D requirements for normal bone growth in puppies may be different from the concentrations required for optimal health and prevention of disease related to insufficiency in adult dogs.

The definition of vitamin D sufficiency in dogs has remained controversial. In man, levels of 25(OH)D from 20–50 ng/ml are required to prevent skeletal abnormalities^(6)^. These levels are controversial due to inconsistent protocols for plasma 25(OH)D determination. Recently, a plasma 25-hydroxyvitamin D3 (25(OH)D_3_) level of 100–120 ng/ml was considered sufficient for dogs, based on the minimisation of variance of parathyroid hormone and mean C-reactive protein levels at this range^(7)^. According to this data, many apparently healthy dogs may be vitamin D insufficient despite most commercial pet foods having adequate vitamin D levels^(8)^. Vitamin D insufficiency in dogs has been associated with a host of various disease processes^(3,7,9–13)^. Whether insufficient vitamin D status is a consequence of disease, or risk factor for disease is presently under investigation. Several studies have indicated a correlation between low vitamin D status and disease, with insufficient vitamin D status corresponding to a poorer prognosis^(7,14,15)^. To combat the potential for deficiency contributing to disease, appropriate supplementation is warranted.

Supplementation with excess vitamin D in the form of cholecalciferol (D3) did not improve plasma D status in privately owned dogs that were considered vitamin D insufficient^(16)^. However, supplementing 25(OH)D_3_ rapidly improved plasma D status in research dogs^(17)^. A similar improvement was found in healthy people, and in people with renal disease^(18–20)^.

The objective of the present study was to determine whether consumption of a diet using vitamin D supplemented as HyD^®^, a proprietary form of 25(OH)D_3_, would increase the plasma 25(OH)D_3_ of healthy dogs above that achievable with D3. Utilising one dog breed served to minimise observational variance from genetic heterogeneity. Golden Retrievers were chosen due to breed predisposition towards low vitamin D status^(2)^, as well as their predisposition to developing various neoplastic diseases and other diseases associated with vitamin D insufficiency^(2,7,21)^. Among dog breeds, Golden Retrievers have a high lifetime risk for the development of cancers^(22)^. This makes them an ideal subject for a study evaluating the improvement of insufficient vitamin D. We hypothesised that supplementation with vitamin D in the form of 25(OH)D_3_ would rapidly and safely increase plasma 25(OH)D_3_ and thereby sustain an elevation in vitamin D status. We also hypothesised that the plasma 25(OH)D_3_ of dogs fed a diet supplemented with a high but not excessive amount of vitamin D3 would not significantly increase.

## Experimental methods

### Part I

#### Animals

All procedures were reviewed and approved by the institutional animal care and use committee (Protocol #8908). Canine participants were recruited at a breed club show (Golden Retriever National Event, St. Louis, Missouri) and an announcement through an e-mail list-serve. A total of fifty-seven privately owned, adult dogs aged 2–10 years were recruited. Dogs were excluded if they were younger than 2 years or older than 10 years, if they had underlying disease processes, were pregnant, lactating, or had pregnancy planned during the trial duration. Dogs were also excluded if they were receiving vitamins or supplements that could impact vitamin D status. Signed informed consent forms were obtained from owners prior to dog evaluation and blood collection. This form relayed the purpose and expected outcomes of the study, and potential risks to the patient. Owners were informed of the potential for their dog to be enrolled in the second part of the study, pending vitamin D status.

#### Evaluation and sample collection

All dogs sampled were purebred Golden Retrievers (thirty-three males, twenty-four females). Body weights ranged from 23⋅5 to 40⋅1 kg. All weights were evaluated on a single scale. Approximately 6 ml of venous blood was obtained from each dog via jugular or cephalic venipuncture. Blood was immediately transferred to a lithium heparinised tube and spun down in a centrifuge at 1200 g for 10 min within 30 min of collection. Plasma was harvested and stored in a separate plastic snap-cap tube in a −20°C freezer until evaluation. Plasma 25(OH)D_3_ status of each animal was evaluated using a high-performance liquid chromatography (HPLC) method^(23)^ that was adapted for analysis of dog serum and plasma^(16,17)^.

### Part II

#### Animals

Owners of dogs determined to have plasma 25(OH)D_3_ concentrations less than 50 ng/ml were notified by phone or e-mail and offered participation in the second part of the study: a diet supplementation trial to evaluate the efficacy of vitamin D supplementation using a custom-manufactured, dry-expanded diet (Table 1). Owners of dogs with the lowest vitamin D status were contacted first, due to the association between vitamin D insufficiency and reduced odds of survival^(14,24)^.

**Table 1.** Target and measured as-fed proximate nutrient contents and concentrations of calcium, phosphorus, D vitamers and metabolisable energy of trial diets^a^

Nutrient	D3		HyD^®^	
	Target	Measured	Target	Measured
Crude protein (%^b^)	39 (min)	40⋅3	39 (min)	39⋅5
Crude fat (%)	14 (min)	11⋅7	14 (min)	10⋅7
Crude fibre (%)	3 (max)	nd	3 (max)	nd
Moisture (%)	12 (max)	6⋅9	12 (max)	6⋅6
Calcium (%)	–	1⋅0	–	1⋅0
Phosphorus (%)	–	0⋅9	–	0⋅9
Vitamin D3 (IU/kg)	3250	3630	0	70
25-hydroxyvitamin D3 (μg/kg)	–	<1	16	19
Metabolisable Energy (kcal/kg)	3623	–	3623	–

nd, Not determined.

a Diet ingredients listed by weight, as is, in decreasing order: Chicken by-product meal, maize gluten meal, dried egg product, brewers rice, maize, chicken fat, wheat, natural flavour, sorghum, dried plain beet pulp, potassium chloride, salt, dicalcium phosphate, pea fibre, calcium carbonate, vitamin E supplement, niacin supplement, thiamine mononitrate, calcium pantothenate, vitamin A supplement, mineral oil, pyridoxine hydrochloride, riboflavin supplement, vitamin D3 supplement, vitamin B12 supplement, folic acid, biotin, tocopherols, ferrous sulphate, zinc oxide, manganese oxide, copper sulphate, sodium selenite, cobalt carbonate, ethylenediamine dihydriodide, taurine, choline chloride.

b Weight (g)/diet weight (100 g).

Reviews of medical and dietary history of all participants were conducted to ensure that participating dogs were healthy. Complete diet histories were requested of all participants. Clinical laboratory variables (complete blood count and routine biochemical analyses of plasma and urine) were evaluated prior to participation. Physical examinations were conducted by primary veterinarians to ensure that canine participants were healthy prior to study commencement. Instructions were provided to owners of participating dogs to avoid alterations in the environment and exercise schedule during the supplementation trial.

#### Groups

Dogs were assigned to a control (*n* 9 initial, *n* 8 completion) or treatment group (*n* 10), stratified based on initial plasma 25(OH)D_3_ status. Groups were balanced based on plasma 25(OH)D_3_ concentration, sex, body weight and group size as much as possible (Table 2). Dogs belonging to the same household were assigned to the same group. Owners were blinded as to which group their dogs were assigned.

**Table 2.** Characteristics and vitamin D status of dogs enrolled in the control (D3) and treatment (HyD^®^) groups at the beginning of the diet supplementation trial

	D3		HyD^®^		
	Median	Range	Median	Range	
Number	8	–	10	–	
Sex (female %)	63	–	60	–	NS*
Age (years)	7⋅5	2–10	4⋅0	2–10	NS^†^
Body weight (kg)	28⋅2	23⋅5–31⋅2	26⋅9	24⋅5–36⋅6	NS^†^
Plasma 25-hydroxyvitamin D (ng/ml)	25⋅3	13⋅9–36⋅6	25⋅6	17⋅7–34⋅7	NS^†^

*P* ≤ 0⋅05 is considered significant.

* Not significantly different by *χ*^2^ analysis.

† Not significantly different by Kruskal–Wallis test.

#### Diet

Once groups were assigned, all dogs received one of two diets formulated in compliance with AAFCO dog food nutrient profiles for adult maintenance except for vitamin D3 (Table 1). The control diet provided vitamin D as D3 and the treatment diet provided vitamin D as 25(OH)D_3_ (HyD^®^, DSM Nutritional Products, LLC, New Jersey 07054). Proximate analysis of a random sample of treatment and control diets was performed at an external laboratory (University of Missouri Agricultural Experiment Station Chemical Laboratories). Owners of participating dogs were provided feeding instructions so that dogs consumed the test diet at the caloric equivalence of their historic diet to maintain their body weight. Dogs were transitioned to their new diet over a 7-d period.

#### Plasma collection

Canine participant owners and veterinary clinics listed by the owners were e-mailed instructions on monthly appointments for blood collection. Blood samples were collected by the author (R. K.) or by the participant's primary veterinary clinic, depending on dog location. Samples were centrifuged and plasma was harvested within 1 h of sample collection. Plasma was shipped overnight on ice packs to the university. Upon arrival, plasma was placed in an airtight, freezer-resistant plastic tube and stored for a maximum of 4 months at −20°C for batch analysis of plasma 25(OH)D_3_ concentrations. Tubes were labelled so that the identity of the sample was not revealed to the personnel measuring plasma 25(OH)D_3._ Blood collections were repeated at weeks 4, 8 and 16 for assessment vitamin D status from plasma 25(OH)D_3_ concentrations. Plasma 25(OH)D_3_ analysis was repeated on all plasma samples at completion of the study to reduce inter-assay variance. Upon completion of the trial, at week 16, all dogs had repeated clinical laboratory analysis of blood, plasma and urine.

#### Laboratory analyses

Clinical haematology (Sysmex XT-2000i; Sysmex America, Inc., Lincolnshire, IL), plasma biochemistry (Beckman AU480, Beckman Coulter, Inc., Brea, CA) and urinalyses (Clinitek Status, Siemens Medical Solutions USA, Inc., Malvern, PA) were performed at the University of Missouri Veterinary Medical Diagnostic Laboratory, Columbia, MO. The same analysers were used for all samples. Plasma 25(OH)D_3_ of each animal was evaluated monthly and batch-evaluated at the end of the experiment to minimise variability using the chromatographic method as described earlier.

#### Statistical analysis

Sample size power analyses were performed to determine the number of dogs required in a parallel study to demonstrate the treatment effect. The power analysis assumed a standard deviation of plasma 25(OH)D_3_ between dogs to be 14 ng/ml. The analysis indicated that a minimum of 46 % difference in mean plasma 25(OH)D_3_ concentration between groups, seven dogs would be required to find significance at *α* = 0⋅05 with the power of *β* = 0⋅8.

Statistical analyses were performed using statistical software (SAS^®^ Version 9⋅4; SAS Institute, Cary, NC). Data were tested for normality using skew and kurtosis measures, and visual inspection of plots. Non-normally distributed data was log-transformed. Normally distributed biochemical and haematologic observations were analysed with paired *t* tests. Non-normally distributed data and categorical results were evaluated using Wilcoxon rank-sign testing. A repeated-measures, mixed models ANOVA that employed Tukey–Kramer adjusted *post hoc* multiple comparisons were used to determine the significance of differences between treatment group outcomes at each blood sampling time, i.e. initial and at 1, 2, 3 and 4 months after the D-vitamer supplementations. Urine calcium/creatinine and urine phosphorus/creatinine ratios were evaluated using paired *t* tests. The significance of D-vitamer supplementation type on frequencies of categorical urinalysis findings was determined with Fisher's exact tests. *P*-values ≤ 0⋅05 were considered significant.

## Results

### Initial survey

In the initial cohort of fifty-seven dogs, plasma 25(OH)D_3_ concentrations ranged from 14 to 59 ng/ml. Fifty-three dogs (91⋅4 %) had 25(OH)D_3_ values below 50 ng/ml. Concentrations were normally distributed. Mean and median concentrations were 34 ng/ml. Twenty-six dogs (45 %) had 25(OH)D_3_ concentrations below 33 ng/ml.

### Group parameters

Initial veterinary examinations indicated that all participants were healthy. Initial laboratory parameters evaluated on participants of part II were within the laboratory reference ranges, with few exceptions (Tables 3–5). Sex distribution, body weight, ages and plasma 25(OH)D_3_ concentrations of both groups were similar (Table 2).

**Table 3.** Plasma clinical haematology variable medians and ranges of dogs before and 4 months after diet supplementation with vitamin D3 (*n* 8) or HyD^®^ (*n* 10)

	Supplementation								
	Vitamin D3			HyD^®^			Vitamin D3 *v*. HyD^®^		
	Pre-trial	4 months	P*	Pre-trial	4 months	P*	Pre-trial P	4 months *P*	Reference interval
Leucocytes (X 1000/μl)	9⋅1	9⋅2	0⋅92	8⋅2	8⋅1	0⋅83	0⋅29	0⋅38	4⋅1–14⋅6
	[5⋅9–11⋅0]	[6⋅7–11⋅7]		[5⋅2–11⋅7]	[5⋅2–12⋅2]				
Erythrocytes (X 1 000 000/μl)	7⋅1	6⋅8	0⋅14	7⋅1	6⋅6	0⋅01	0⋅69	0⋅94	5⋅3–8⋅3
	[6⋅2–8⋅2]	[6⋅3–7⋅1]		[5⋅5–8]	[5⋅8–7⋅7]				
Haemoglobin (g/dl)	16⋅6	16⋅3	0⋅24	17	16⋅4	0⋅01	0⋅93	0⋅99	13⋅3–20⋅8
	[15–20⋅6]	[15–17⋅8]		[13⋅7–19⋅7]	[14⋅4–19⋅2]				
Haematocrit (%)	48⋅2	48⋅2	0⋅38	49⋅7	48⋅5	0⋅01	0⋅91	0⋅96	37⋅2–56⋅4
	[42⋅9–61⋅1]	[44⋅2–52⋅2]		[42–57⋅3]	[43⋅5–53⋅3]				
MCV^†^ (fl)	69	73⋅1	0⋅04	70⋅6	71⋅1	0⋅33	0⋅57	0⋅69	62⋅5–72⋅9
	[66⋅8–74⋅8]	[66⋅6–77⋅5]		[67⋅3–76⋅9]	[67⋅9–81⋅4]				
MCHgb^‡^ (pg)	24⋅2	25⋅0	0⋅08	24⋅6	24⋅7	0⋅73	0⋅45	0⋅96	22⋅4–26⋅2
	[22⋅9–25⋅8]	[22⋅9–26]		[23⋅3–26⋅4]	[23⋅5–26⋅4]				
MCHC^§^ (g/dl)	34⋅3	34⋅2	0⋅21	34⋅5	34⋅1	0⋅35	0⋅76	0⋅51	34⋅2–37⋅9
	[33⋅7–35⋅5]	[33⋅3–34⋅6]		[32⋅6–36⋅1]	[32⋅5–36⋅6]				
Platelets (X 1000/μl)	283	289	0⋅15	256	278	0⋅23	0⋅39	0⋅47	140–350
	[220–399]	[247–465]		[174–450]	[150–477]				
Segmented neutrophils (X 1000/μl)	5⋅21	5⋅45	0⋅96	4⋅65	4⋅30	0⋅65	0⋅32	0⋅24	2⋅27–10⋅60
	[3⋅11–7⋅72]	[3⋅21–7⋅63]		[2⋅79–8⋅05]	[2⋅14–8⋅19]				
Band neutrophils (X 1000/μl)	0⋅06	0⋅12	0⋅63	0⋅03	0⋅04	0⋅49	0⋅36	0⋅24	0⋅00–0⋅18
	[0⋅00–0⋅28]	[0⋅00–0⋅35]		[0⋅00–0⋅09]	[0⋅00–0⋅17]				
Lymphocytes (X 1000/μl)	2⋅70	2⋅58	0⋅94	2⋅34	2⋅67	0⋅24	0⋅40	0⋅67	0⋅83–4⋅80
	[1⋅88–3⋅14]	[1⋅4–3⋅62]		[1⋅3–3⋅72]	[2⋅06–4⋅97]				
Monocytes (X 1000/μl)	0⋅30	0⋅31	0⋅59	0⋅20	0⋅21	0⋅56	0⋅12	0⋅35	0⋅05–1⋅24
	[0⋅18–1⋅10]	[0⋅20–0⋅58]		[0⋅10–0⋅60]	[0⋅10–0⋅64]				
Eosinophils (X 1000/μl)	0⋅31	0⋅39	0⋅28	0⋅45	0⋅45	0⋅54	0⋅46	0⋅63	0⋅07–1⋅40
	[0⋅11–0⋅98]	[0–1⋅17]		[0⋅08–1⋅33]	[0⋅21–1⋅06]				
Basophils (X 1000/μl)	0	0		0	0				nr^†^
	[0–0]	[0–0]		[0–0]	[0–0]				
Nucleated erythrocytes	0	0		0	0				0–1
	[0–0]	[0–0]		[0–1]	[0–1]				

* *P*-values of comparisons between pre-trial and month 4 values.

† MCV, Mean Corpuscular volume.

‡ MCHgb, mean corpuscular haemoglobin.

§ MCHC, Mean Corpuscular Haemoglobin Concentration.

**Table 4.** Plasma clinical chemistry variable medians and ranges of dogs before and 4 months after diet supplementation with vitamin D3 (*n* 8) or HyD^®^ (*n* 10)

	Supplementation								
	Vitamin D3			HyD^®^			Vitamin D3 *v*. HyD^®^		
	Pre-trial	4 months	P*	Pre-trial	4 months	P*	Pre-trial *P*	4 months *P*	Reference interval
Glucose (mol/l)	5⋅5	5⋅4	0⋅95	5⋅4	5⋅1	0⋅48	0⋅34	0⋅17	4⋅5–6⋅4
	[5–6⋅3]	[4⋅6–5⋅7]		[4⋅6–5⋅7]	[4⋅9–5⋅7]				
Urea nitrogen (mg/dl)	16	21	0⋅01	17	20	0⋅01	0⋅45	0⋅21	8–29
	[13–24]	[17–25]		[7–20]	[14–24]				
Creatinine (mg/dl)	1⋅0	0⋅9	0⋅03	1⋅0	0⋅8	0⋅01	0⋅40	0⋅39	0⋅7–1⋅4
	[0⋅7–1⋅2]	[0⋅7–0⋅9]		[0⋅8–1⋅3]	[0⋅7–0⋅9]				
Sodium (meq/l)	147	147	0⋅62	147	147	0⋅07	0⋅79	0⋅13	145–151
	[145–149]	[146–154]		[147–149]	[136–149]				
Potassium (meq/l)	4⋅2	4⋅0	0⋅82	4⋅1	3⋅8	0⋅34	0⋅13	0⋅18	3⋅5–4⋅9
	[3⋅9–4⋅4]	[3⋅6–4⋅9]		[3⋅6–4⋅3]	[3⋅4–4⋅3]				
Chloride (meq/l)	112	113	0⋅56	113⋅5	113	0⋅57	0⋅22	0⋅87	110–117
	[110–116]	[111–116]		[112–116]	[106–117]				
Bicarbonate (meq/l)	22	19⋅5	0⋅01	20	17⋅5	0⋅01	0⋅25	0⋅16	17–26
	[17–26]	[14–21]		[18–24]	[15–20]				
Anion Gap (meq/l)	17	19	0⋅01	18	20	0⋅01	0⋅46	0⋅56	12–20
	[14–19]	[17–31]		[14–19]	[16–21]				
Albumin (g/dl)	3⋅0	3⋅0	1⋅00	3⋅1	3⋅1	0⋅34	0⋅37	0⋅74	2⋅7–3⋅7
	[2⋅8–3⋅2]	[2⋅7–3⋅4]		[2⋅8–3⋅2]	[2⋅7–3⋅2]				
Total Protein (g/dl)	5⋅7	6⋅1	0⋅51	5⋅85	6⋅0	0⋅49	0⋅62	0⋅39	5⋅4–6⋅9
	[5⋅5–6⋅6]	[5⋅5–6⋅8]		[5⋅5–6⋅4]	[5⋅4–9⋅6]				
Globulin (g/dl)	2⋅8	3⋅3	0⋅01	2⋅8	3⋅0	0⋅12	0⋅74	0⋅16	2⋅4–3⋅7
	[2⋅5–3⋅6]	[2⋅4–3⋅8]		[2⋅4–3⋅2]	[2⋅5–3⋅4]				
Calcium (mg/dl)	9⋅55	9⋅7	0⋅16	9⋅65	9⋅85	0⋅47	0⋅34	0⋅56	9⋅1–10⋅8
	[9⋅3–9⋅8]	[9⋅5–10⋅8]		[9⋅2–10⋅2]	[9⋅3–10⋅5]				
Phosphorus (mg/dl)	3⋅8	3⋅2	0⋅02	3⋅8	3⋅5	0⋅76	0⋅22	0⋅14	2⋅3–5⋅0
	[3⋅4–4⋅4]	[2⋅7–3⋅5]		[2⋅1–4⋅3]	[2⋅5–4⋅0]				
Cholesterol (mg/dl)	217	252	0⋅03	245	294	0⋅18	0⋅40	0⋅51	131–320
	[208–249]	[236–351]		[186–403]	[215–488]				
Total Bilirubin (mg/dl)	0⋅2	0⋅2	0⋅63	0⋅2	0⋅2	1⋅00	0⋅52	1⋅00	0⋅1–0⋅4
	[0⋅1–0⋅2]	[0⋅1–0⋅3]		[0⋅2–0⋅3]	[0⋅2–0⋅3]				
ALT^†^ (units/l)	35	34	0⋅94	29	38	0⋅49	0⋅53	0⋅85	14–76
	[17–166]	[23–115]		[17–52]	[16–152]				
ALP^‡^ (units/l)	23	15	0⋅17	23	15	0⋅15	0⋅78	0⋅74	12–98
	[10–28]	[9–30]		[12–32]	[6–28]				
GGT^§^ (units/l)	3	3	1⋅00	3	3	1⋅00	0⋅59	0⋅22	0–8
	[3–4]	[3–3]		[3–4]	[3–8]				
CK^ǁ^ (units/l)	64	97⋅5	0⋅04	59⋅5	65	0⋅79	0⋅96	0⋅05	40–226
	[40–109]	[48–411]		[41–156]	[39–116]				

* *P*-values of comparisons between pre-trial and month 4 values.

† Alanine aminotransferase.

‡ Alkaline phosphatase.

§ γ-glutamyl transferase.

ǁ Creatine kinase.

**Table 5.** Clinical laboratory urinalysis results of dogs before and 4 months after diet supplementation with vitamin D3 (*n* 8) or HyD^®^ (*n* 10)

		Supplementation					
		Vitamin D3		HyD^®^		Vitamin D3 *v*. HyD^®^	
		Pre-trial	4 months	Pre-trial	4 months	Pre-trial *P*	4 months *P*
Specific gravity	Mean Range	1⋅032 [1⋅012–1⋅064]	1⋅032 [1⋅005–1⋅053]	1⋅032 [1⋅004–1⋅059]	1⋅038 [1⋅015–1⋅054]	0⋅98	0⋅61
Glucose	Negative (*n*)	7	8	10	10	nd*	nd
	≥ Trace (*n*)	0	0	0	0		
Bilirubin	Negative/1+ (*n*)	7	8	9	9	1⋅00	1⋅00
	≥2+ (*n*)	0	0	1	1		
Ketones	Negative/trace (*n*)	7	8	10	10	nd	nd
	> Trace (*n*)	0	0	0	0		
Heme	Negative/trace (*n*)	7	8	10	9	nd	1⋅00
	≥1+ (*n*)	0	0	0	1		
pH	Median Range	6⋅0 [5⋅5–7⋅5]	8⋅5 [5⋅5–8⋅5]	6⋅5 [5⋅5–8⋅5]	6⋅8 [5⋅5–8⋅5]	0⋅49	0⋅26
Protein by dipstick	Negative (*n*)	6	5	10	9	0⋅41	0⋅27
	≥1+ (*n*)	1	3	0	1		
Urobilinogen	0⋅2	7	8	10	10	nd	nd
(EU^†^/dL)	>0⋅2	0	0	0	0		
							
Leucocyte^‡^/hpf^§^	None/1–3 (*n*)	6	7	9	6	1⋅00	0⋅31
	>3 (*n*)	1	1	1	4		
Erythrocyte^ǁ^/hpf	None/rare (*n*)	7	7	10	9	nd	1⋅00
	≥1 (*n*)	0	1	0	1		
Bacteria/hpf	None (*n*)	7	8	10	9	nd	1⋅00
	Few (*n*)	0	0	0	1		
Transitional Cells/lpf^¶^	None	None	None	None	None	nd	nd
Squamous Cells/lpf	None to 1–5 (*n*)	7	8	10	9	nd	1⋅00
	>5 (*n*)	0	0	0	1		
Casts/lpf	None (*n*)	7	8	10	9	nd	1⋅00
	Rare (*n*)	0	0	0	1		
Crystals/lpf	None (*n*)	6	5	8	7	1⋅00	1⋅00
	Moderate/many (*n*)	1	3	2	3		
	Struvite (*n*)	1	3	2	2		
	Other (*n*)	0	0	0	1^a^		

* Not determined because of lacking observation in one category.

† Ehrlich units.

‡ Leucocytes.

§ High-power field.

ǁ Erythrocytes.

¶ Low-power field.

a One dog with ‘many struvites and moderate calcium oxalate dehydrate’ which had very concentrated urine.

### Laboratory values

Clinical haematology and biochemistry values and urinalyses parameters at the trial end were not significantly different between groups. There were a few significant differences between pre- and post-trial values within groups (Tables 3–5). At the trial end, the means of erythrocyte counts, haemoglobin concentrations and haematocrits of dogs of the HyD^®^ group decreased (*P* < 0⋅01) slightly by 7, 4 and 2 %, respectively. The means of erythrocyte counts and haemoglobin concentrations of dogs of the vitamin D group by the trial end were also less (−4 and −2 %, respectively) but not significantly. No changes were indicative of adverse health effects and the majority of median values were within the reference interval. Cholesterol and plasma alanine aminotransferase (ALT) values of several dogs were elevated outside the reference interval but were not associated with clinical signs in the dogs.

### Plasma 25(OH)D_3_ status

**Fig. 1.**
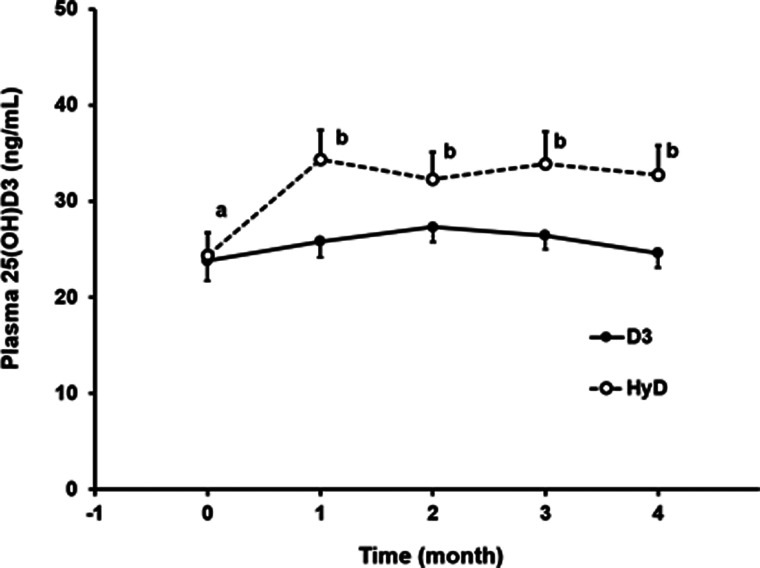
Plasma 25(OH)D_3_ concentrations over time for control (D3) and treatment (HyD^®^) groups. All values are means ± sems. Log-transformed data of means of 25(OH)D_3_ concentrations were compared using repeated-measures ANOVA. Plotted values with different letters indicate significant differences (*P* < 0⋅05).

Plasma 25(OH)D_3_ did not differ between groups at the beginning of the study (Table 2). At Part II initiation, canine participants had plasma 25(OH)D_3_ levels between 13. 9 and 36⋅6 ng/ml. Dogs receiving diet supplemented with HyD^®^ had an increase in plasma 25(OH)D_3_ from a mean of 24⋅4 ng/ml to a mean of 34⋅3 ng/ml (*P* < 0⋅05) after 1 month (Fig. 1). Plasma 25(OH)D_3_ concentrations did not significantly change after the initial increase. At the trial end, plasma 25(OH)D_3_ concentrations in dogs that received the HyD^®^ diet were increased in all but one dog and ranged from a low of 24 ng/ml to a high of 47 ng/ml. The repeated-measures mixed-model ANOVA when applied with a contrast testing without including initial observations revealed a diet effect (*P* = 0⋅044). Mean plasma 25(OH)D_3_ concentrations for dogs (±sem) supplemented with HyD^®^ was 33⋅3 (±3⋅0 ng/ml) which was 28 % greater than the mean 25(OH)D_3_ concentration of dogs supplemented with D3 (26⋅0 ± 1⋅5 ng/ml) (Table 6). For dogs given a diet supplemented with D3, no significant change occurred in plasma 25(OH)D_3_ concentrations. For these dogs, plasma 25(OH)D_3_ concentrations at the study end ranged from a low of 19 ng/ml to a high of 32 ng/ml.

**Table 6.** Statistical significance of effects of supplementation type (HyD^®^ *v*. D3), time of sampling (month), and interaction of effects (HyD^®^ *v*. D3 × month) on log-transformed plasma 25-hydroxyvitamin D3 concentrations (ng/ml)

Effect	Degrees of freedom		*F*-value	*P*
	Numerator	Denominator		
HyD^®^ *v*. D3	1	64	2⋅75	0⋅1022
Month (initial, 1, 2, 3 4)	4	64	13⋅76	<0⋅0001
HyD^®^ *v*. D3 × Month	4	64	4⋅24	0⋅0042
HyD^®^ *v*. D3^a^	NA	64	NA	0⋅0047

a Baseline removed from values.

### Adverse effects

There were rarely reported adverse events during the trial. Inappetence following antibiotic treatment of a urinary tract infection occurred in a dog that received the D3-supplemented diet. The dog was subsequently voluntarily withdrawn from the trial. An undesired change in coat colour and consistency was reported by an owner of a dog receiving diet supplemented with HyD^®^. This change resolved during the trial. Elevation of ALT concentration above the upper limit of the reference interval was revealed via serum biochemistry performed at the trial end in one dog fed the diet supplemented with HyD^®^. Follow-up biochemistry analysis of this dog showed a reduction of ALT to within the reference interval after 4 months post-trial. One owner reported improvement in stool quality while the dog was fed the D3-supplemented diet. No other notable responses were reported.

## Discussion

We hypothesised that dogs fed a diet supplemented with 25(OH)D_3_ would experience a rapid improvement in vitamin D status. This effect was anticipated based on previous research in other species, coupled with findings of an acute rise in plasma 25(OH)D_3_ when purpose-bred dogs were fed treats supplemented with 25(OH)D_3_^(17,25)^. In our study, an initial mixed-model ANOVA did not reveal any significant difference in plasma 25(OH)D_3_ between the dog groups. This result likely reflected the inclusion of 25(OH)D_3_ observations at time point ‘0’ in the analysis, during which the dogs had not yet received their assigned experimental diets. This was anticipated as the treatment groups were balanced based on preliminary plasma 25(OH)D_3_ concentration. A significant group effect was observed once the initial time point was omitted from the statistical analysis (Table 6).

An immediate effect of ingested 25(OH)D_3_ on plasma 25(OH)D_3_ was anticipated for several reasons. Absorption of D3 relative to 25(OH)D_3_ occurs more slowly, by passive diffusion, largely from intestinal micelles, and possibly through intestinal epithelial cholesterol transporter activity^(26)^. Sufficient amounts of luminal fat, intestinal lipase activity and bile acid concentration are required to form needed micelles. Absorbed D3, which principally enters intestinal lymph free or esterified and incorporated in lipoproteins or bound to plasma proteins, is then sequestered by the liver, adipose, and other tissues^(27–29)^. From the liver, D3 is gradually released after 25-hydroxylation to 25(OH)D_3_. Comparatively, alimentary uptake of 25(OH)D_3_ is more rapid than that of D3 because it has greater water solubility and intestinal protein-binding capabilities^(30,31)^. Alimentary 25(OH)D_3_ enters circulation more directly relative to D3, first in portal blood then systemic circulation bound to plasma proteins^(32)^.

Dogs do not respond to supplementation of D3 in high amounts to improve vitamin D status^(16,17)^. Furthermore, dogs and man with liver or gastrointestinal disease have been found insufficient in vitamin D and may have impairments in vitamin D metabolism that could lower their D status^(9,33,34)^. For these patients, supplementation with 25(OH)D_3_ may be ideal, due to its increased bioavailability to counteract reduced vitamin D metabolism capabilities.

Another substantive finding was no significant change in plasma 25(OH)D_3_ concentration for the dogs consuming diet supplemented with D3. The D3 content of the experimental diet (approximately 1000 IU/Mcal, Table 1) was in excess of the current recommended maximum by AAFCO (750 IU/Mcal)^(35)^. It is less than the prior maximum (1250 IU/Mcal), which several years ago was reduced to the current value to be in accord with NRC safe-upper-limit and FEDIAF Guidelines. The D3 concentration in the experimental diet was likely greater than that of diets dogs were consuming prior to the study, when time 0 plasma 25(OH)D_3_ concentrations were determined. Kritkos *et al.* conducted a survey of eighty-one commercially available dog foods and reported a median vitamin D content of 481 IU/Mcal, with no foods in excess of the current 750 IU/Mcal^(8)^. The D3 content of the experimental diet was comparatively high for dog foods, suggesting that the dogs maintained on the experimental diet were resistant to change in vitamin D status. Heaney *et al.* suggest that as liver 25-hydroxylases of man become saturated with the D3 substrate, the production of 25(OH)D_3_ by the liver becomes progressively less influenced by increasingly available D3^(29)^. Hence, resistance to change in vitamin D status with oral vitamin D supplementation should be expected if liver 25-hydroxylase activity is near maximal.

While circulating 25(OH)D_3_ concentration is a widely accepted indicator of vitamin D status for many species, specific concentrations of 25(OH)D_3_ that indicate vitamin D deficiency, insufficiency, sufficiency and excess are only tentatively agreed upon. An international consensus report on vitamin D status of man recently suggested that serum concentrations of 25(OH)D_3_ less than 12 ng/ml (30 nmol/l) indicate vitamin D deficiency, while concentrations between 20 ng/ml (50 nmol/l) and 50 ng/ml (125 nmol/l) indicate vitamin D sufficiency for skeletal health^(6)^. Vitamin D status sufficient for skeletal health maybe insufficient for other health outcomes^(36)^. For example, serum 25(OH)D_3_ concentrations between 30 and 40 ng/ml (75–100 nmol/l) may reduce colorectal cancer risk in man^(37)^. Threshold 25(OH)D_3_ concentrations and limits for extra-skeletal disease prevention and clinical interventions largely have not been established, although they are being investigated^(6)^. Use of varying methods for analysis of 25(OH)D_3_ have impeded progress; a ‘gold standard’ methodology is not established. In general, chromatographic methods are less affected by the sample matrix and permit independent quantification D3 and D2 metabolites, and are therefore considered superior to immunoassays^(38)^.

Selting *et al.* used a competitive chemiluminescence immunoassay in their work and reported serum 25(OH)D_3_ concentrations of vitamin D sufficiency in dogs to be 100–120 ng/ml. Our 25(OH)D_3_ concentrations were lower than theirs. The concentration difference likely reflected assay methodological differences, more than vitamin D deficiency in our dogs. A comparison of results obtained for thirty-three dog serum samples by our HPLC method with the method used by Selting *et al.* indicates the competitive chemiluminescence immunoassay 25(OH)D_3_ values are on average greater by about 100 % (R. C. B., unpublished results). In using chromatographic methods similar to those used in our study, Horst and Littldike found 25(OH)D_3_ circulates in many domesticated species between 30 and 50 ng/ml^(39)^. Our analyses show 25(OH)D_3_ concentrations ranged from 14 to 59 ng/ml and had a median value of 34 ng/ml^(39)^. The aforementioned consensus regarding circulating 25(OH)D concentrations in man, if speculatively applied to our observations in dogs, indicates that many dogs that we evaluated in the present study, i.e. dogs with 25(OH)D_3_ concentrations less than 20 ng/ml, would be considered vitamin D ‘insufficient’.

Low initial plasma 25(OH)D_3_ concentrations in some dogs might have reflected low dietary vitamin D intake, although the vitamin D contents of diets historically consumed by the dogs were not determined. At the study end, two dogs that received a diet supplemented with D3 had plasma 25(OH)D_3_ concentrations just below 20 ng/ml. For these dogs, 25-hydroxylation of D3 to form 25(OH)D_3_ might have been limited. None of the dogs fed the diet supplemented with 25(OH)D_3_ had plasma 25(OH)D_3_ concentrations below 20 ng/ml by the study end. Low vitamin D status of individual dogs, lines of dogs within a breed or dogs of a breed in general as has been reported for immature Great Danes could be rooted in the variation of 25-hydroxylase activity. Liver and kidney microsomal and mitochondrial enzymes with 25-hydroxylase activity (CYP2R1, CYP3A4 and CYP27A) are believed to synthesise 25(OH)D_3_ from D3^(40,41)^. Some polymorphisms of genes encoding the hydroxylases have functional consequences involving vitamin D metabolism in man^(42)^. Our findings on dietary 25(OH)D_3_ supplementation demonstrate a means to raise the vitamin D status of dogs that might have low vitamin D status despite consuming recommended amounts of D3. Besides circumvention of need for 25(OH)D_3_ synthesis (e.g. as with some liver disease), ingestion of 25(OH)D_3_ presents a means to correct vitamin D insufficiency resulting from malabsorption and/or maldigestion of fat that leads to low vitamin D status, as might occur in dogs with inflammatory bowel disease^(9,33,34)^.

The use of HyD^®^ for supplementation was chosen for its demonstrated safety and effectiveness in livestock feeds. HyD^®^ has been safely and effectively used in poultry feeds for more than 20 years as a vehicle for 25(OH)D_3_ supplementation. Dietary 25(OH)D_3_ in place of D3 improves early skeletal development and immune function in broiler chickens^(43)^. Using HyD^®^ in swine feeds has been described more recently. Benefits to skeletal health of sows are inferred from findings and greater birth weights of piglets are observed^(44)^. The amount of 25(OH)D_3_ used in supplementation was much less than that of D3. Our results indicate that the potency of 25(OH)D_3_ in the HyD^®^ supplement was more than five times that of D3 for the dietary matrix studied. A greater potency of 25(OH)D_3_ relative to D3 for raising plasma 25(OH)D_3_ concentration is consistent with prior work in dogs and dosage trials in other species^(17,32,43,45,46)^.

The HyD^®^ supplementation was well-tolerated in the present study. Clinical haematology and chemistry results were not significantly different with supplementation type; however, subsequent changes may have occurred with a longer study duration. Although significant changes from pre-trial values occurred for some variables, most changes were modest, within clinical laboratory reference intervals (Table 4), and not considered to affect the health of the dogs. The majority of haematologic values were within the reference range (Table 3), with the exception of mean corpuscular volume, which was not considered to be clinically significant. The modest declines in observed haemoglobin, haematocrit and erythrocytes might have reflected environmental exposures of the dogs. The trial was initiated during late fall and winter and concluded during spring and early summer. In captive wolves, declines from winter to summer are reported in haemoglobin, haematocrit, erythrocytes and mean corpuscular haemoglobin concentration^(47)^. The mechanism is speculative but may involve endocrine factors like circulating thyroxine which is found to be greater in winter than in summer in wolves. Biochemical median values were all within reference ranges. Urinalyses were not significantly different, all of which indicated the safety of the diets used. Owners reported no signs of serious health effects. The cause for plasma alanine ALT elevation in one dog that received the HyD^®^ diet is unknown. The elevation was not severe and corrected after the dietary change.

Limitations of the present study are important to note. Participants were supplemented for 4 months, and haematologic and biochemical values were not evaluated for most participants once the dogs were transitioned off of the study diet. It is possible that sequelae of vitamin D supplementation occurred following transition off of the study diet. This is less likely given the half-life of 25(OH)D_3_ appears to be 1⋅8 weeks in dogs^(17)^. Even though one breed of dog was used, significant inter-animal variation was sufficient to reduce the group effect, justifying the limitation to one breed. The dogs used in the study were healthy dogs. As previously stated, it is unclear whether vitamin D insufficiency is associated with disease development or contributes to it. Therefore, results may differ when using dogs with various disease processes. While the subjects in this trial were healthy, confirming that a consistent and safe rise in vitamin D status with HyD^®^ supplementation in healthy dogs is an essential first step prior to supplementing patients with concurrent disease processes. Follow-up studies are essential to determine the efficacy of supplementation with HyD^®^ in improving vitamin D status in individuals with disease processes associated with vitamin D insufficiency.

## References

[1] How KL, Hazewinkel HA & Mol JA (1994) Dietary vitamin D dependence of cat and dog due to inadequate cutaneous synthesis of vitamin D. Gen Comp Endocrinol 96, 12–18. 7843559 10.1006/gcen.1994.1154

[2] Sharp CR, Selting KA & Ringold R (2015) The effect of diet on plasma 25-hydroxyvitamin D concentrations in dogs. BMC Res Notes 8, 442. 26374201 10.1186/s13104-015-1360-0 PMC4570747

[3] Stocklin E & Eggersdorfer M (2013) Vitamin D, an essential nutrient with versatile functions in nearly all organs. Int J Vitam Nutr Res 83, 92–100. 24491882 10.1024/0300-9831/a000151

[4] Cartwright JA, Gow AG, Milne E, (2018) Vitamin D receptor expression in dogs. J Vet Intern Med 32, 764–774. 29469965 10.1111/jvim.15052 PMC5866978

[5] National Research Council (2006) Nutrient Requirements of Dogs and Cats. Washington, DC: The National Academies Press. 10.17226/10668.

[6] Giustina A, Adler RA, Binkley N, (2020) Consensus statement from 2nd International Conference on Controversies in Vitamin D. Rev Endocr Metab Disord 21, 89–116. 32180081 10.1007/s11154-019-09532-w PMC7113202

[7] Selting KA, Sharp CR, Ringold R, (2016) Plasma 25-hydroxyvitamin D concentrations in dogs – correlation with health and cancer risk. Vet Comp Oncol 14, 295–305. 25041357 10.1111/vco.12101

[8] Kritikos G, Weidner N, Atkinson JL, (2018) Quantification of vitamin D3 in commercial dog foods and comparison with Association of American Feed Control Officials recommendations and manufacturer-reported concentrations. J Am Vet Med Assoc 252, 1521–1526. 29889635 10.2460/javma.252.12.1521

[9] Gow AG, Else R, Evans H, (2011) Hypovitaminosis D in dogs with inflammatory bowel disease and hypoalbuminaemia. J Small Anim Pract 52, 411–418. 21797872 10.1111/j.1748-5827.2011.01082.x

[10] Kraus MS, Rassnick KM, Wakshlag JJ, (2014) Relation of vitamin D status to congestive heart failure and cardiovascular events in dogs. J Vet Intern Med 28, 109–115. 24205918 10.1111/jvim.12239 PMC4895547

[11] Gerber B, Hassig M & Reusch CE (2003) Plasma concentrations of 1,25-dihydroxycholecalciferol and 25-hydroxycholecalciferol in clinically normal dogs and dogs with acute and chronic renal failure. Am J Vet Res 64, 1161–1166. 13677396 10.2460/ajvr.2003.64.1161

[12] Wakshlag JJ, Rassnick KM, Malone EK, (2011) Cross-sectional study to investigate the association between vitamin D status and cutaneous mast cell tumours in Labrador retrievers. Br J Nutr 106, S60–S63. 22005438 10.1017/S000711451100211X

[13] Mick PJ, Peng SA & Loftus JP (2019) Plasma vitamin D metabolites and CXCL10 concentrations associate with survival in dogs with immune mediated disease. Front Vet Sci 6, 247. 31417914 10.3389/fvets.2019.00247 PMC6682597

[14] Jaffey JA, Backus RC, McDaniel KM, (2018) Plasma vitamin D concentrations in hospitalized critically ill dogs. PLoS ONE 13, e0194062. 29590167 10.1371/journal.pone.0194062 PMC5874018

[15] Titmarsh HF, Gow AG, Kilpatrick S, (2015) Low vitamin D status is associated with systemic and gastrointestinal inflammation in dogs with a chronic enteropathy. PLoS ONE 10, e0137377. 26333093 10.1371/journal.pone.0137377 PMC4557950

[16] Young LR & Backus RC (2016) Oral vitamin D supplementation at five times the recommended allowance marginally affects plasma 25-hydroxyvitamin D concentrations in dogs. J Nutr Sci 5, e31. 27547394 10.1017/jns.2016.23 PMC4976120

[17] Young LR & Backus RC (2017) Plasma 25-hydroxyvitamin D_3_ and 24R,25-dihydroxyvitamin D_3_ concentrations in adult dogs are more substantially increased by oral supplementation of 25-hydroxyvitamin D_3_ than by vitamin D_3_. J Nutr Sci 6, e30. 29152235 10.1017/jns.2017.8 PMC5672302

[18] Bischoff-Ferrari HA, Dawson-Hughes B, Stocklin E, (2012) Oral supplementation with 25(OH)D_3_ versus vitamin D3: effects on 25(OH)D_3_ levels, lower extremity function, blood pressure, and markers of innate immunity. J Bone Miner Res 7, 160–169. 10.1002/jbmr.551 22028071

[19] Cashman KD, Seamans KM, Lucey AJ, (2012) Relative effectiveness of oral 25-hydroxyvitamin D3 and vitamin D3 in raising wintertime serum 25-hydroxyvitamin D in older adults. Am J Clin Nutr 95, 1350–1356. 22552038 10.3945/ajcn.111.031427

[20] Heaney RP, Davies KM, Chen TC, (2003) Human plasma 25-hydroxycholecalciferol response to extended oral dosing with cholecalciferol. Am J Clin Nutr 77, 204–210. 12499343 10.1093/ajcn/77.1.204

[21] Schiffman JD & Breen M (2015) Comparative oncology: what dogs and other species can teach us about humans with cancer. Phil Trans R Soc B 370, 20140231. 26056372 10.1098/rstb.2014.0231 PMC4581033

[22] Torres de la Riva G, Hart BL, Farver TB, (2013) Neutering dogs: effects on joint disorders and cancers in golden retrievers. PLoS ONE 8, e55937. 23418479 10.1371/journal.pone.0055937 PMC3572183

[23] Lensmeyer GL, Wiebe DA, Binkley N, (2006) HPLC method for 25-hydroxyvitamin D measurement: comparison with contemporary assays. Clin Chem 52, 1120–1126. 16574756 10.1373/clinchem.2005.064956

[24] Cazzolli DM, Prittie JE, Fox PR, (2019) Evaluation of serum 25-hydroxyvitamin D concentrations in a heterogeneous canine ICU population. J Vet Emerg Crit Care 29, 605–610. 10.1111/vec.12901 31637855

[25] Dusso A, Lopez-Hilker S & Rapp N (1998) Extra-renal production of calcitriol in chronic renal failure. Kidney Int 34, 368–375. 10.1038/ki.1988.190 3172645

[26] Silva MC & Furlanetto TW (2018) Intestinal absorption of vitamin D: a systematic review. Nutr Rev 76, 60–76. 29025082 10.1093/nutrit/nux034

[27] Fraser DR & Kodicek E (1968) Enzyme studies on the esterification of vitamin D in rat tissues. Biochem J 109, 457–467. 4301039 10.1042/bj1090457 PMC1186839

[28] Tsuprykov O, Chen X, Hocher CF, (2018) Why should we measure free 25(OH) vitamin D? J Steroid Biochem Mol Bio 180, 87–104. 29217467 10.1016/j.jsbmb.2017.11.014

[29] Heaney RP, Armas LAG, Shary JR, (2008) 25-Hydroxylation of vitamin D3: relation to circulating vitamin D3 under various input conditions. Am J Clin Nutr 87, 1738–1742. 18541563 10.1093/ajcn/87.6.1738

[30] Stamp TCB (1974) Intestinal absorption of 25-hydroxycholecalciferol. Lancet 2, 121–123. 4135557 10.1016/s0140-6736(74)91553-0

[31] Teegarden D, Nickel KP & Shi L (2000) Characterization of 25-hydroxvitamin D binding protein from intestinal cells. Biochem Biophys Res Commun 275, 845–849. 10973809 10.1006/bbrc.2000.3397

[32] Cesareo R, Falchetti A, Attanasio R, (2019) Hypovitaminosis D: is it time to consider the use of calcifediol? Nutrients 11, 1016. 10.3390/nu11051016 PMC6566727 31064117

[33] Allenspach K, Rizzo J, Jergens AE, (2017) Hypovitaminosis D is associated with negative outcome in dogs with protein losing enteropathy: a retrospective study of 43 cases. BMC Vet Rest 13, 96. 10.1186/s12917-017-1022-7 PMC5385077 28390394

[34] Khan MA, Dar HA, Baba MA, (2019) Impact of vitamin D status in chronic liver disease. J Clin Exp Hepatol 9, 574–580. 31695247 10.1016/j.jceh.2019.03.001 PMC6823692

[35] Association of American Feed Control Officials Incorporated. AAFCO Official Publication. C2020. Chapter 4. Model Regulations for Pet Food and Specialty Pet Food Under the Model Bill. P141–224.

[36] Holick MF (2017) The vitamin D deficiency pandemic: approaches for diagnosis, treatment and prevention. Rev Endocr Metab Disord 18, 153–165. 28516265 10.1007/s11154-017-9424-1

[37] McCullough ML, Zoltick ES, Weinstein SJ, (2019) Circulating vitamin D and colorectal cancer risk: an international pooling project of 17 cohorts. J Natl Cancer Inst 111, 158–169. 29912394 10.1093/jnci/djy087 PMC6376911

[38] Binkley N & Carter GD (2017) Toward clarity in clinical vitamin D status assessment: 25(OH)D assay standardization. Endocrinol Metab Clin North Am 46, 885–899. 29080641 10.1016/j.ecl.2017.07.012

[39] Horst RL & Littledike ET (1982) Comparison of plasma concentrations of vitamin D and its metabolites in young and aged domestic animals. Comp Biochem Physiol B Biochem Mol Biol 73, 485–489. 10.1016/0305-0491(82)90064-5 6983948

[40] Tryfonidou MA, Holl MS, Vastenburg M, (2003) Hormonal regulation of calcium homeostasis in two breeds of dogs during growth at different rates. J Anim Sci 81, 1568–1580. 12817506 10.2527/2003.8161568x

[41] Jolliffe DA, Walton RT, Griffiths CJ, (2016) Single nucleotide polymorphisms in the vitamin D pathway associating with circulating concentrations of vitamin D metabolites and non-skeletal health outcomes: review of genetic association studies. J Steroid Biochem Mol Biol 164, 18–29. 26686945 10.1016/j.jsbmb.2015.12.007

[42] Duan L, Xue Z, Ji H, (2018) Effects of CYP2R1 gene variants on vitamin D levels and status: a systematic review and meta-analysis. Gene 678, 361–369. 30120973 10.1016/j.gene.2018.08.056

[43] Litta G (2016) Applications of 25OHD_3_ in poultry meat production. CAB Rev 11, 1–10.

[44] Weber GM, Witschi AK, Wenk C, (2014) Triennial growth symposium–effects of dietary 25-hydroxycholecalciferol and cholecalciferol on blood vitamin D and mineral status, bone turnover, milk composition, and reproductive performance of sows. J Anim Sci 92, 899–909. 24492559 10.2527/jas.2013-7209

[45] Vaes AMM, Tieland M, de Regt MF, (2018) Dose-response effects of supplementation with calcifediol on serum 25-hydroxyvitamin D status and its metabolites: a randomized controlled trial in older adults. Clin Nutr 37, 808–814. 28433267 10.1016/j.clnu.2017.03.029

[46] Graeff-Armas LA, Bendik I, Kunz I, (2020) Supplemental 25-hydroxycholecalciferol is more effective than cholecalciferol in raising serum 25-hydroxyvitamin D concentrations in older adults. J Nutr 150, 73–81. 31518424 10.1093/jn/nxz209

[47] Seal US & Mech LD (1983) Blood indicators of seasonal metabolic patterns in captive adult gray wolves. J Wildl Manage 47, 704–715.

